# Assessments of Secondary Reinforcement of Epoxy Matrix-Glass Fibre Composite Laminates through Nanosilica (SiO_2_)

**DOI:** 10.3390/ma11112186

**Published:** 2018-11-05

**Authors:** Prince Jeya Lal Lazar, Ramesh Sengottuvelu, Elango Natarajan

**Affiliations:** 1 Department of Mechanical Engineering, KCG College of Technology, Chennai 600 097, India; ramesh_1968in@yahoo.com; 2 Faculty of Engineering, UCSI University, Kuala Lumpur 56000, Malaysia; cad.elango.n@gmail.com

**Keywords:** nanosilica, epoxy matrix, E-glass fibre, X-ray diffraction, SEM, C-Scan

## Abstract

The principal objective of this research work was to investigate the results of impregnating epoxy matrix-glass fibre composite laminates with nanosilica as secondary reinforcement. 0.5, 0.75, 1 and 3 wt% nanosilica was used and thereafter properties of composites were assessed through tensile, three point bending, quasi static indentation tests and dynamic mechanical analysis. Scanning electron microscope examinations were done on fracture surfaces and failure modes were analyzed. The internal failures of the composite due to quasi-static indentation were evaluated through C-Scan. Among samples of different weight fractions, 0.75 wt% nanosilica reinforced composite laminates exhibited substantial increase of 42% in tensile strength and 39.46% in flexural strength. The reduction in glass transition temperature (T_g_), increase in storage modulus (E′), loss modulus (E″) and damping factor (tan δ) were also observed. Quasi-static indentation assessments revealed that energy absorption property was enhanced significantly by 53.97%. Hence nanosilica up to 0.75 wt% can be used as a potential candidate for secondary reinforcement in epoxy composite laminates.

## 1. Introduction

The need and applications of polymer composites had significantly increased in the recent decades due to its superior properties, stiffness and strength to weight ratio. Engineers prefer them for marine, aerospace and automobile applications since polymer composites display enhanced fatigue and corrosion resistant properties and moreover, it can be tailored to a specific requirement of the customer [1,2]. Despite all these merits, polymer composites possess their own drawbacks which have been addressed by many researchers. Okoli and Smith [3] highlighted brittle failure of polymer matrix which results in poor yielding, poor load transfer to fibers due to weak interfacial bonding of fiber-matrix, fracture of fibers and delamination. Choi et al. [4] revealed that initial brittle failure of matrix is the root cause for other failure mechanisms in polymer composites under low velocity impact. Akoi et al. [5] observed the similar kind of failure mechanism under quasi-static indentation. Furthermore, defects may occur due to accumulation of residual stress during curing after different types of loadings. Since matrix materials possess high cross link density, the decrease in toughness was observed in various cases as reported by [6,7].

These limitations can be overcome through structural modification of matrix with reinforcements of nano size which would result in enhanced stiffness of composite material [8,9]. Initially, researchers used micro fillers like CaCO_3_, Ceramics, Alumina, Fly ash, Clay, Mica, etc. as reinforcements. Nano fillers have the potential to enhance the properties of composites drastically than micro fillers for various engineering applications [10,11]. Reinforcing the polymer matrix with nano fillers like, single walled carbon nanotubes (SWCNT), double walled carbon nanotubes (DWCNT) and multi walled carbon nanotubes (MWCNT) [12,13,14,15], graphene [16,17,18] and nano-clay [19,20,21] were attempted. Significant enhancement in the performance of materials was reported after reinforcing nano fillers. Zhen-Kun et al. [22] reported that significant increase in the tensile and impact strength was observed in cryogenic testing conditions by reinforcing MWCNTs. Zhang et al. [23] reported enhanced fracture toughness in epoxy by using nanosilica as a secondary reinforcement. However, researchers encountered setbacks like agglomeration and homogeneous dispersion of nano fillers in matrix [24]. This could be due to the fact that, specific surface area of nano fillers were much higher than micro fillers and were in the range of 20 to 250 m^2^/g. Moreover, agglomeration of nanofillers is much more complex phenomena and the large surface area might be only one of the reasons. It is to be noted that nano particles reinforced polymer composites with enhanced mechanical properties is achievable only by ensuring optimum dispersion of nano fillers in the matrix. Techniques like three roll milling, ultra-sonication, solution mixing and calendering are reported in literature [25,26,27] that addresses homogeneous dispersion of nano fillers in matrix. Liao et al. [28] and Montazeri et al. [29] also explained the technique to ensure optimum dispersion of nano fillers and enhancement of properties through nano fillers. Elango et al. [30] used Fourier Transform Infrared Spectroscopy (FTIR), SEM and Atomic Force Microscopy (AFM) to ensure the dispersion of MWCNTs over polymer matrix. 

Nanosilica is identified as a new potential candidate material for secondary reinforcement as it occupies the tiniest voids and enhances the interfacial binding of the fibers in the laminates which results in substantial stress transfer. A few research activities using nanosilica as secondary reinforcement is reported in [31,32,33]. Gong et al. [31] synthesized silica nano fillers of size 80 nm by sol gel technique. They successfully achieved homogeneous dispersion using mechanical blending and reported enhanced interfacial bonding between glass fibre and modified epoxy. Fathy et al. [32] synthesized silica nano particle of size 10–20 nm and investigated the fatigue behaviour of nano silica reinforced glass fibre composite laminates. Johnsen et al. [33] studied the effect of nano fillers of different sizes as toughening agents and explored the associated toughening mechanisms. However sufficient literature does not exist in relation to failure behaviour and internal damage behaviour of nanosilica reinforced composites.

The objective of the present research was to impregnate glass fiber epoxy composite laminates with nanosilica as secondary reinforcement and to investigate its effect on the tensile, flexural, quasi static indentation and visco-elastic properties. The properties of the resultant composite laminates were examined conferring to American Society for Testing and Materials (ASTM) standards. The failure surfaces were examined through SEM and internal damage induced due to quasi-static indentation was investigated through an advanced nondestructive evaluation (NDE) technique. 

## 2. Materials and Methods

Plain weave E-Glass Fabric in mat form and medium viscosity, unmodified epoxy resin based on Diglycidyl Ether of Bisphenol A (DGEBA) Epoxy LY556 along with the Hardener HY951 were used as primary reinforcement and matrix respectively. Secondary reinforcement material is hydrophilic nanosilica with the average particle size of 17 nm. All the materials were procured from Sakhti Fiber Glass Limited, Chennai, Tamil Nadu, India. The properties of primary reinforcement, matrix and secondary reinforcement are presented in the Table 1 and Table 2.

### 2.1. Preparation of Nano Composites

**Figure 1:**
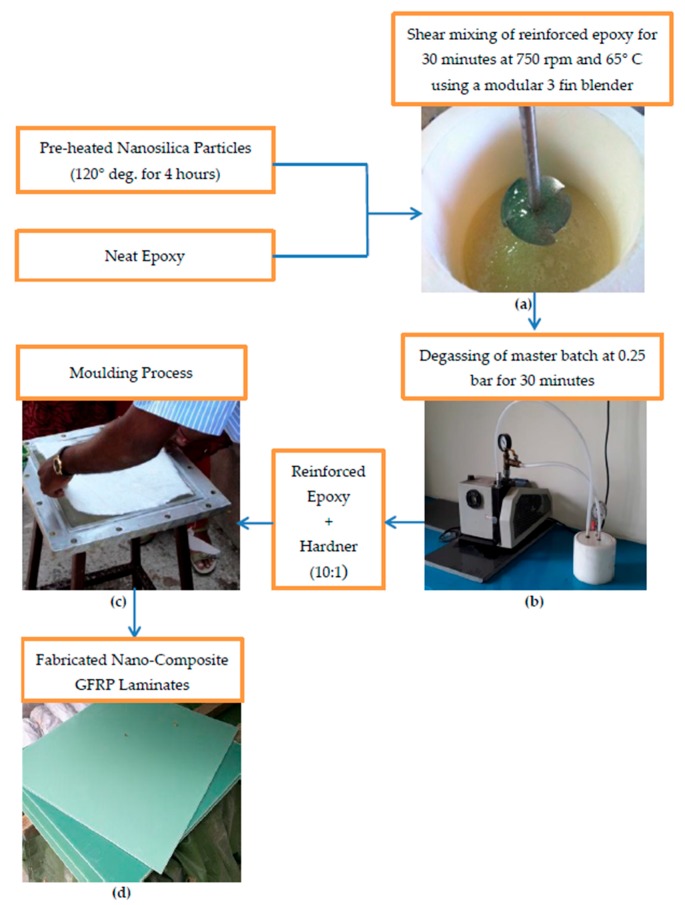
Manufacturing scheme of nanosilica reinforced GFRP laminates. (**a**) Shear mixing of epoxy and nanosilica (**b**) Degassing of modified epoxy using vacuum chamber kit (**c**) Hand layup process (**d**) View of cured nanocomposite laminates.

Composite laminates of 3 ± 0.2 mm thick were prepared by arranging six layers of plain weave glass fiber mat one over the other inside a mould. Matrix was reinforced with nanosilica in varying weight ratio of 0.5, 0.75, 1 and 3 wt%. Homogeneous dispersion of nanosilica in matrix was attained by mechanical blending as shown in Figure 1a for 30 minutes at 750 rpm and 65 °C using a modular 3 fin blender. This master batch was then placed in a vacuum chamber Figure 1b and degased at 0.25 bar to eliminate all the air bubbles generated during mixing. Elimination of such defects had an effect on the mechanical properties of the composite laminate. The homogeneous mix of modified matrix-hardner was prepared in the ratio 10:1 and was applied on each layer of glass fiber mat as shown in Figure 1c. Finally, the mould was compressed to squeeze excess resin out and it was then allowed to cure for 24 h under compression. Figure 1 depicts the overall manufacturing scheme of nanosilica reinforced glass fiber reinforced plastic (GFRP) laminates. It is to be noted that curing nature of the matrix is influenced by reinforcing nano fillers. Total quantity of resin required for the process was estimated using following expression.

$$W_{R}=\frac{W_{F}}{70}\%\;\times \;40\%$$

where,

*W_R_* is the weight of resin required (g).

*W_F_* is the weight of the glass fibre (g).

The fiber fraction of composite laminates was estimated as 69.23% from burn off test as per ASTM D3171. A computer numerical control (CNC) operated abrasive water jet cutting machine was used to cut the samples as per standards from the laminate since delamination, fiber pull-outs and structural defects may occur in conventional jig saw cutting technique. The cutting parameters were set as suggested in [34]. The composite laminates produced by the compression moulding technique displayed minimum voids and good surface quality during visual evaluation.

### 2.2. Characterization Studies

All the experiments were conducted as per ASTM Standards at a National Accreditation Board for Laboratories (NABL) Accredited and National Accreditation Board for Certification Bodies (NABCB) Accredited National Laboratory in Chennai, Tamil Nadu, India.

#### 2.2.1. Tensile and Flexural Tests

Tensile tests were carried out in universal tensile testing machine (Instron 3382 with 100 kN load cell) at room temperature (23 °C, 50% humidity) as per ASTM D3039. A wedge type grip was used to hold and prevent the specimen from slipping and breakage of specimen at grip region. The tensile load was gradually applied to the sample at a crosshead speed of 5 mm/min and the responses such as tensile strength, tensile modulus and failure strain of the sample were recorded. Five samples in each weight fraction were examined and the mean of the readings were computed and considered.

Flexural tests were carried out on samples as per ASTM D7264 using an Autograph AG-IS equipment (Shimadzu, Tokyo, Japan) fitted with 50 kN load cell and an especially designed holding module to hold the sample. The speed of the crosshead was set to 2 mm/min. The three-point bending load was applied at the middle of the sample and the responses like flexural strength, flexural modulus and displacement to failure were recorded as per standards. 

#### 2.2.2. Quasi Static Indentation Tests

Quasi static indentation tests as per ASTM D6264 were carried out in Instron 3382 universal testing machine (Instron, Norwood, MA, USA) fitted with a 100 kN load cell and a hemispherical indenter along with an especially designed fixture. The diameter of the hemispherical indenter was 12.7 mm. Laminates were clamped rigidly to the fixture and indentation force was exerted exactly at the centre of the rectangular specimen at a displacement rate of 5 mm/min. Test responses like compressive stress, peak contact force, stiffness and displacement of indenter with respective time values were recorded online using Bluehill software (V2, Instron, Norwood, MA, USA). 

#### 2.2.3. XRD Analysis

XRD study is one of the best tools to recognize the structure of nano composites. Information in regard to the degree of crystallinity, molecular arrangements and polymorphism can be understood from XRD. Nano scale sized crystals are observed in polymers along the thickness direction and its large surface to volume ratio enhances interfacial disorganization. XRD analysis was carried out on samples for different weight fraction of nanosilica in order to understand the degree of dispersion of secondary reinforcement in matrix. The powder form of samples was analyzed in XRD Machine (Bruker D8, Bruker AXS GmbH, Karlsruhe, Germany) operated at 40 kV, 40 mA and at a scanning rate of 2°/min. The crystallographic spacing (d-spacing) was estimated by Bragg’s law. The nano composite structure (exfoliated or intercalated) was evaluated based on the modified values of d-spacing.

#### 2.2.4. Dynamic Mechanical Analysis

Dynamic Mechanical Analysis (DMA) is a primary tool for rapid characterization of the visco-elastic behaviour of polymeric materials. DMA was carried out in TA Q800 V20.6 Build24 (TA Instruments, New Castle, DE, USA) as per ASTM D4065 to understand the visco-elastic behaviour of nanocomposites. The analysis was carried out in dual cantilever mode with temperature sweep from 30 °C to 180 °C at a heating rate of 5 °C/min. A constant low frequency of 1 Hz was set in order to avoid the break in polymer chains. Storage modulus (E′), loss modulus (E″) and damping factor (tan δ) as a function of temperature (t) were recorded. By evaluating the results of DMA, the influence of secondary reinforcement on the design variables like stiffness, glass transition temperature and energy dissipation behaviour were understood.

### 2.3. Failure Analysis

#### 2.3.1. Scanning Electron Microscopy

Interfacial bonding analysis and fracture analysis were carried out using Carl Zeiss SUPRA-55 Variable Pressure Field Emission Scanning Electron Microscope (ZEISS, Oberkochen, Germany) that is operating at extremely high voltages (from 0.2 kV) and capable of producing high magnification images (<10,000,000,000×). Prior to analysis, all the test samples were coated with a layer of gold having a thickness about 20 nm to avoid the accumulation of electrons.

#### 2.3.2. Non-Destructive Evaluation Using C-Scan

Internal damages induced by quasi-static indentation tests were analyzed using an Olympus make immersion ultrasonic transducer (Olympus Corporation, Tokyo, Japan) with a focal length of 0.8 inch operated at 1 MHz in pulse echo mode. JSR make DPR300 (Imaginant Inc., New York, NY, USA) was used to generate and process the signals. The composite laminate was placed on a fixture which was completely immersed in water, as water has desirable reflection and transmission coefficient values when compared to air. The transducer was held using a mechanical manipulator and was used to manage the translations of the transducer in x, y and z directions. For precise measurements, gain was set as 47 dB and scanning grid steps were set in the range of 0.4 mm for longitudinal and transverse directions with a standoff distance 0.8 inch. Customized interfacing software acqUT & extUT (Indian Institute of Technology Madras, Chennai, India) was used to control the motion of the manipulator and the signals received from the specimens were processed to record A-Scan, B-Scan and C-Scan results separately.

## 3. Results and Discussions

### 3.1. Characterisation Results

#### 3.1.1. XRD Analysis

**Figure 2:**
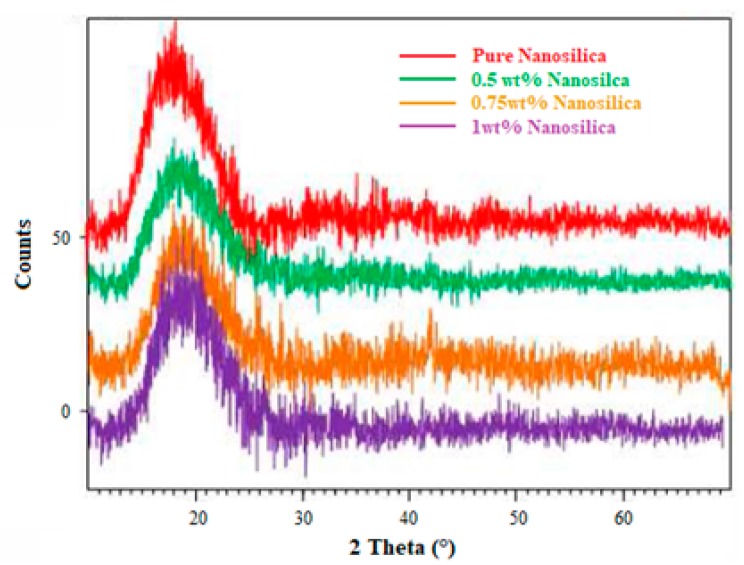
XRD pattern of pure and modified composites.

Dispersion of filler in matrix may be termed as either intercalated or exfoliated in regard to the interlayer distance. If the (d-spacing) interlayer distance is estimated higher than 10 nm, then the dispersion behaviour is termed as exfoliated [35]. Figure 2 shows the XRD pattern of pure and nano silica reinforced composite laminates. The degree of dispersion of nanosilica in the matrix was vividly noticed in XRD. The neat epoxy registered an intense peak at 18.99° with a high slope whereas, 1, 0.75 and 0.5 wt% nanosilica reinforced composite laminates registered intensity of peak at 18.55°, 18.59° and 18.97° respectively. The intensity of peak is noticed to decrease as the filler weight percentage increases. The d-spacing of nanosilica reinforced composite laminates is noticed significantly increased from 4.27 Å to 4.76 Å for 0.75 wt% filler reinforced composite as compared to other weight fractions. This ensured enhanced dispersion of nanosilica in epoxy and an intercalated structure as reported by [36]. Very weak peaks were recorded in the case of 1 wt% of nanosilica due to the exfoliated structure. This could be due to the build-up of viscosity and shear-thinning of the matrix with the increase in nanosilica content, which ended up in poor dispersion and high agglomeration. 

#### 3.1.2. Dynamic Mechanical Analysis (DMA) Response

**Figure 3:**
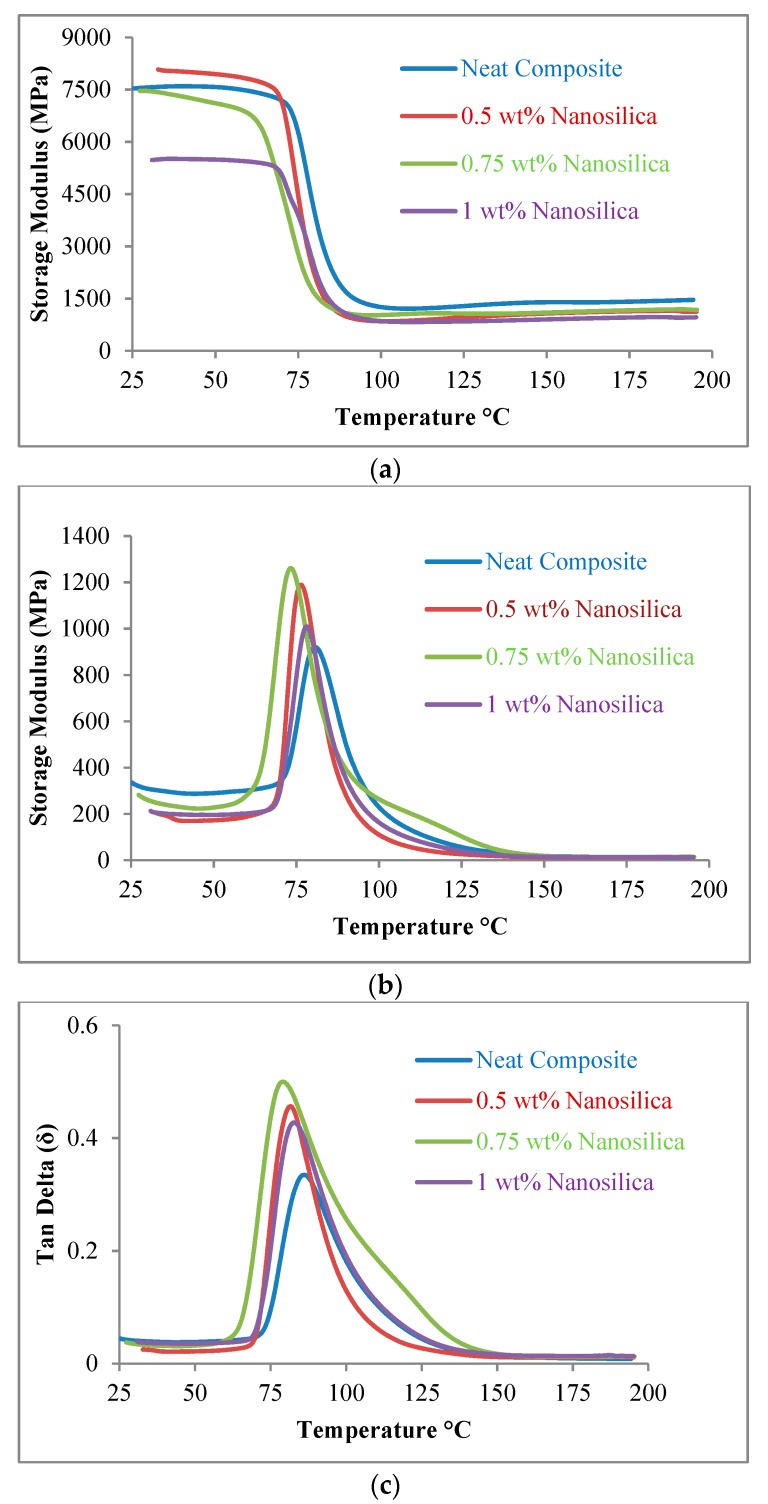
Visco-elastic properties of neat and nanocomposites (**a**) Storage modulus; (**b**) Loss modulus; (**c**) Damping behaviour.

The visco-elastic properties of neat composites and nanocomposites for different weight fractions of secondary reinforcement are displayed in Figure 3 and Table 3. 

***Storage Modulus.*** An increase in the storage modulus (E′) for 0.5 wt% nanosilica reinforced composite laminates was witnessed in the elastic region at ambient temperature. A drop in storage modulus (E′) was witnessed for 0.75 wt% and 1 wt% nanosilica composites at ambient temperature as presented in Figure 3a. This could be due to the impregnation of nano fillers which resulted in the increase of stiffness for 0.5 wt% nanocomposites. However, a plateau was observed in the temperature ranging from 100–200 °C. The continued increase of temperature above 90 °C enhanced molecular movements in polymer chains and hence the laminates exhibited a purely ductile behaviour. At elevated temperatures, laminates exhibited minimum stiffness and high flexibility. Lowest value of storage modulus was registered for the highly loaded (1 wt%) composites in both glassy and rubbery regimes. With higher nanosilica concentration (1 wt%), stiffness enhanced and composites exhibited brittle nature. Stiffness reduced rapidly at 73.02 °C, 69.83 °C, 63.61 °C and 68.72 °C for 0, 0.5, 0.75 and 1 wt% samples respectively. These were considered as glass transition temperatures (T_g_).

***Loss Modulus.*** Neat composites registered a loss modulus of 920 MPa. Whereas, 0.5 wt% (DMA-L2) and 0.75 wt% (DMA-L3) nanosilica reinforced composites registered loss modulus of 1188 MPa and 1261 MPa respectively. DMA-L3 exhibited a decent increase in loss modulus by 37.06% and DMA-L2 exhibited an increase by 29.13% when compared to neat composite. Highly loaded nanocomposites recorded unsatisfactory results. A shift in loss modulus peaks towards the lower temperature was observed in Figure 3b ensuring the enhancement in the agility of polymer chains. It was also observed that, the loss modulus curve was spread over a greater area for the laminate DMA-L3 when compared to neat and other nanocomposite samples. This could be due to the enhanced interfacial bonding between fibre and matrix and enhanced stress transfer at the ply interface. This resulted in improved energy dissipation through internal friction between molecular chains. The same fact was also evidenced in SEM studies which are discussed in the following section. 

***Damping.***Figure 3c illustrates the damping behaviour of neat and nanosilica reinforced composite laminates. Damping factor of neat composite laminates was assessed as 0.334. In case of nanocomposites, damping factor was assessed as 0.455, 0.499 and 0.426 with respect to DMA-L2, DMA-L3 and DMA-L4 laminates. Damping factor (tan δ) was enhanced by 36.22%, 49.4% and 27.54% for DMA-L2, DMA-L3 and DMA-L4 laminates respectively. DMA-L3 recorded superior damping behavior due to the enhanced energy dissipation through impregnated secondary reinforcements in matrix. Modified composites with nanosilica (DMA-L3) registered highest value of tan δ as witnessed by a broader curve than neat composite. This was due to the generation of micro cracks and enhanced energy transfer to primary fiber reinforcements through the secondary nano reinforcement particles.

#### 3.1.3. Tensile Response

**Figure 4:**
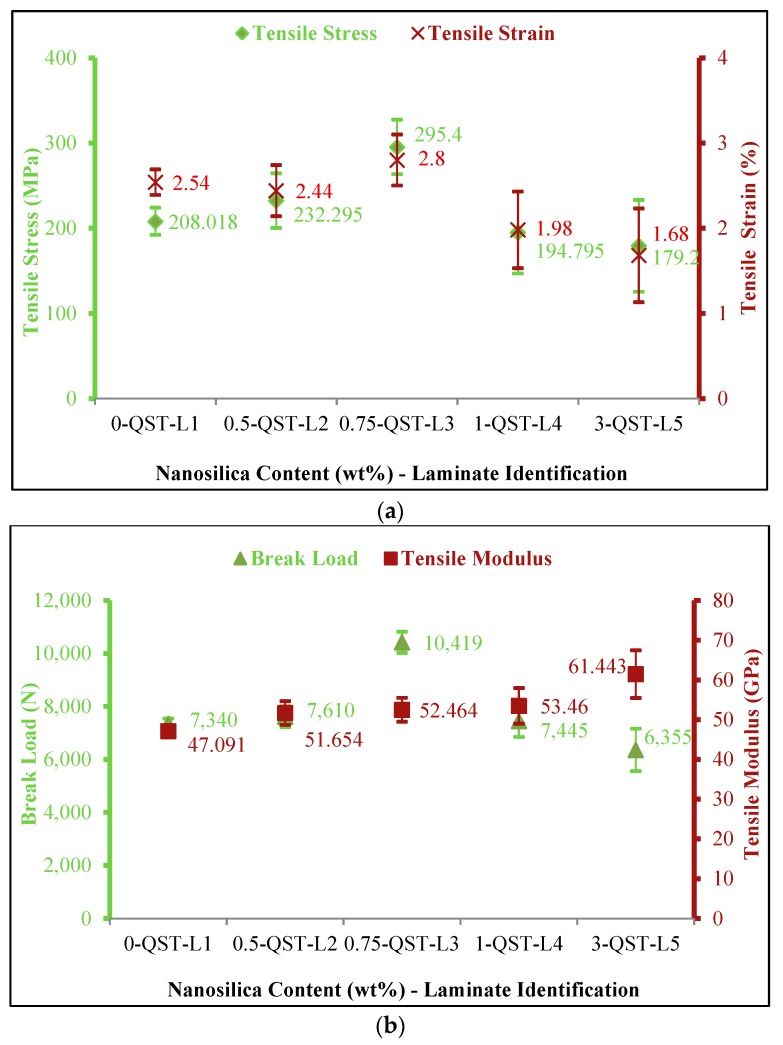
Tensile properties of nanocomposites (**a**) stress-strain behaviour of nanocomposites; (**b**) breaking load and tensile modulus of nanocomposites.

Figure 4 illustrates the tensile properties of nanocomposites. It is interesting to note that, tensile strength, which is a fibre centered property, was recorded as 208.018 MPa for neat composites. Whereas, tensile strength was in the order of 232.295 MPa, 295.4 MPa, 194.795 MPa and 179.2 MPa for 0.5, 0.75, 1 and 3 wt% nanosilica concentrations respectively. Composite laminates with 0.75 wt% nanosilica content (QST-L3) recorded substantial rise in tensile strength which is 42% higher than neat composites. With the increase in nanosilica content, a steady rise in tensile modulus was recorded for all cases. Neat composites exhibited a tensile modulus of 47.091 GPa. Whereas, tensile modulus was recorded as 51.654 GPa, 52.464 GPa, 53.86 GPa and 56.443 GPa for 0.5, 0.75, 1 and 3 wt% nanosilica concentrations respectively. Tensile modulus increased by 11.4% in case of (QST-L3) when compared to neat composites. Likewise, for QST-L2, 9.68% increase in tensile modulus was recorded. This increase in tensile strength was due to the homogenous dispersion of nano particles in matrix as evidenced by XRD studies and enhanced load transfer at the fiber matrix interface. However, decrease in tensile properties due to enhanced stiffness and matrix embrittlement in polymer composites reinforced with 1 and 3 wt% nanosilica (QST-L4 and QST-L5) as reported by [21,37]. It is understood that the melt viscosity of the matrix increased with higher loading of fillers (≥1 wt%) that correspondingly leads to matrix embrittlement, poor wetting of matrix on primary and secondary and poor load transfer between fiber and matrix. This poor dispersion of fillers through the matrix lead to agglomerations, defected weak regions and voids that created localized failure stresses.

#### 3.1.4. Three Point Bending Response

**Figure 5:**
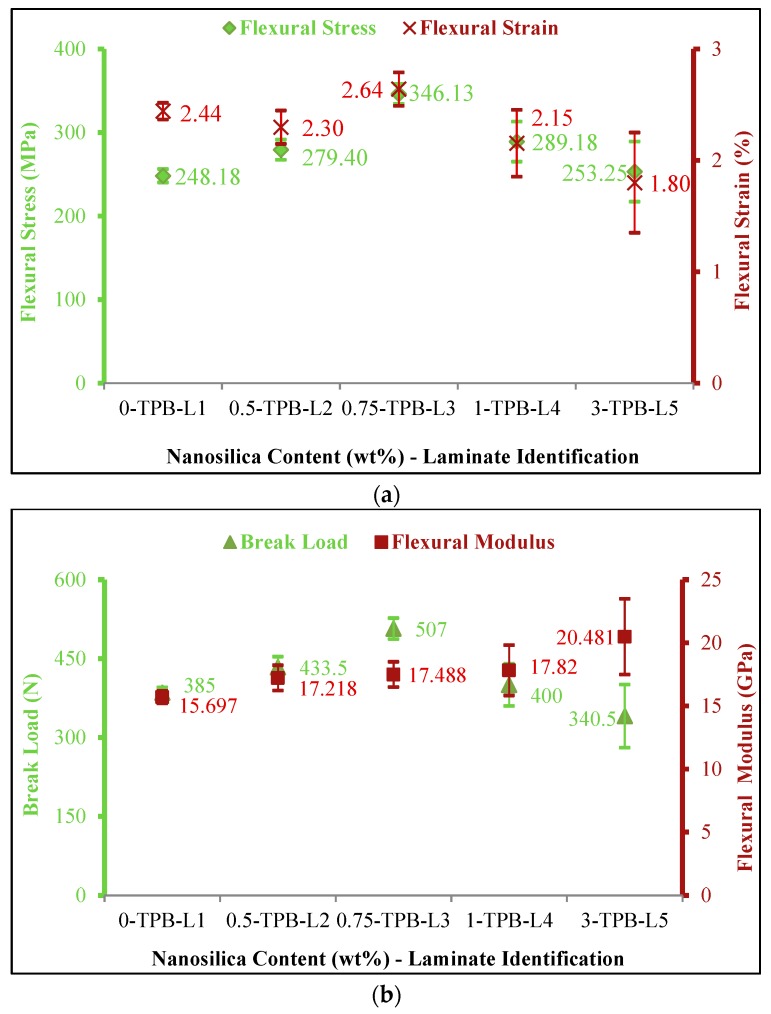
Three points bending properties of nanosilica reinforced composites (**a**) stress-strain behaviour of nanocomposites; (**b**) breaking load and flexural modulus of nanocomposites.

Brittle failure of matrix, delamination at ply interface and failure of fibers were witnessed during the experiments. Though failure occurred due to the combination of tensile, compression and shear loads, compression of the top surface lead to matrix failure which was the predominant reason of failure in three point bending. Figure 5 illustrates the flexural behaviour of nanocomposites. A remarkable escalation in flexural strength was noted with the rise in secondary reinforcement. Flexural strength was in the order of 248.18 MPa, 279.40 MPa, 346.13 MPa, 289.18 MPa and 253.25 MPa for 0, 0.5, 0.75, 1 and 3 wt% nanocomposites respectively. A substantial enhancement of flexural strength by 39.46% was observed in case of 0.75 wt% of nanosilica reinforced composites (TPB-L3). A gradual increase in the flexural strength in the order of 2.04%, 16.5% and 12.57% was observed for the laminates TPB-L5, TPB-L4 and TPB-L2 respectively. Neat composites exhibited a flexural modulus of 15.697 GPa. Whereas, flexural modulus was recorded as 17.218 GPa, 17.488 GPa, 17.82 GPa and 20.481 GPa for 0.5, 0.75, 1 and 3 wt% nanosilica reinforced composites respectively. Flexural modulus displayed a decent increase of 11.4% in TPB-L3. Likewise, flexural modulus recorded improvement in the order of 30.47%, 13.52% and 9.68% for the laminates TPB-L5, TPB-L4 and TPB-L2 respectively. The obtained results were in similar trend to the results reported by [21,37].

#### 3.1.5. Quasi Static Indentation Test Results

**Figure 6:**
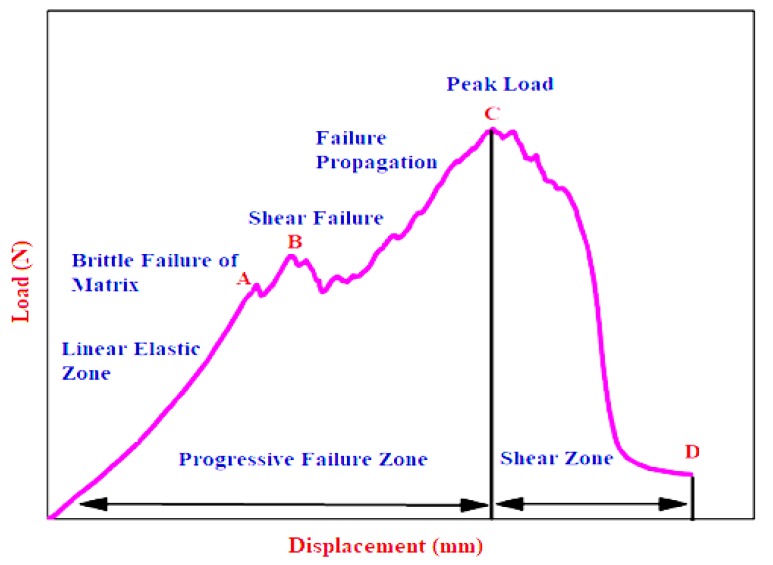
Stages of failure in quasi-static indentation analysis.

A typical force-displacement of fiber reinforced composite laminates subjected to quasi-static indentation is represented in Figure 6, elucidating the various stages of failure. Energy absorbed by the laminate during indentation was estimated by calculating the area under the force-displacement curve. While the composite laminate was subjected to compressive loading, a linear rise in contact force was observed until the point A where the elastic behaviour of material ends which was evidenced by a cracking sound of matrix and plastic failure of matrix-fibre follows. Thereafter, progressive failure of matrix and fiber occurred until it had reached the peak force at C. Maximum damage occurred between B and C which was the plastic region.

**Figure 7:**
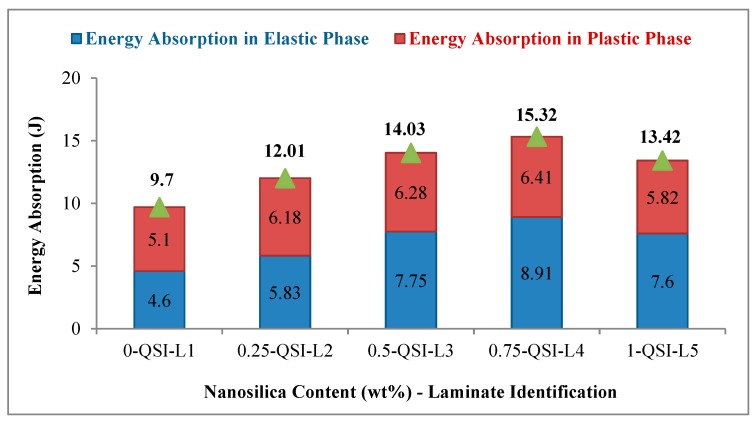
Energy absorption behaviour in elastic and plastic regime of neat and nano-composites.

The responses of neat and nanocomposites under quasi static indentation are presented in Table 4. It was noted that the indentation properties of the laminates were enhanced steadily with the addition of secondary reinforcements. Figure 7 represents the energy absorbed in elastic and plastic phase of composite laminates with varying weight fraction of the secondary reinforcement. 

Total energy absorbed by the composite laminates during compression was estimated by quantifying the area below the force-displacement curve. The quasi-static indentation properties of neat composite and nanosilica reinforced composites and the energy absorbed by nano-composite laminates until it reached the peak compressive force (peak load) is presented in Table 4. The laminate QSI-L4 exhibited highest energy absorption in both elastic region (8.91 J) and plastic region (6.41 J). Highest time 85.3 s was recorded by QSI-L4 composite laminate to attain the peak compressive force, thus revealing that it absorbed more energy in progressive failure phase. It was noted that the region between initial matrix failures to peak load (A-C) increased significantly in QSI-L4 when compared to neat composites. This could be due to the enhanced stress distribution and stress transferring ability of nano sized secondary reinforcements embedded in matrix. The addition of nano fillers reformed the energy absorbing behaviour of composites. QSI-L3 also recorded convincing results when compared to neat composites. QSI-L4 recorded 44.46% increase in energy absorption under elastic region and 59.75% increase in plastic region when compared to neat polymer composites QSI-L1. In regard to total energy absorption behaviour till peak load, QSI-L4 absorbed 15.32 J and recorded a noteworthy increase of 57.93% when compared to QSI-L1. QSI-L3 recorded a decent increase of 44.63% when compared to QSI-L1. These results were superior to the results published by [38]. A decent increase of 33.47%, 19.61% and 33.5% was recorded by QSI-L4 in terms of compressive stress, stiffness and peak load respectively compared with QSI-L1. Energy absorption of QSI-L5 (high loaded composite) was not promising due to the agglomeration of nanosilica, which was evidenced in tensile testing as well.

### 3.2. Failure Analysis

#### 3.2.1. Fiber-Matrix Interfacial Bonding and Fracture Analysis

**Figure 8:**
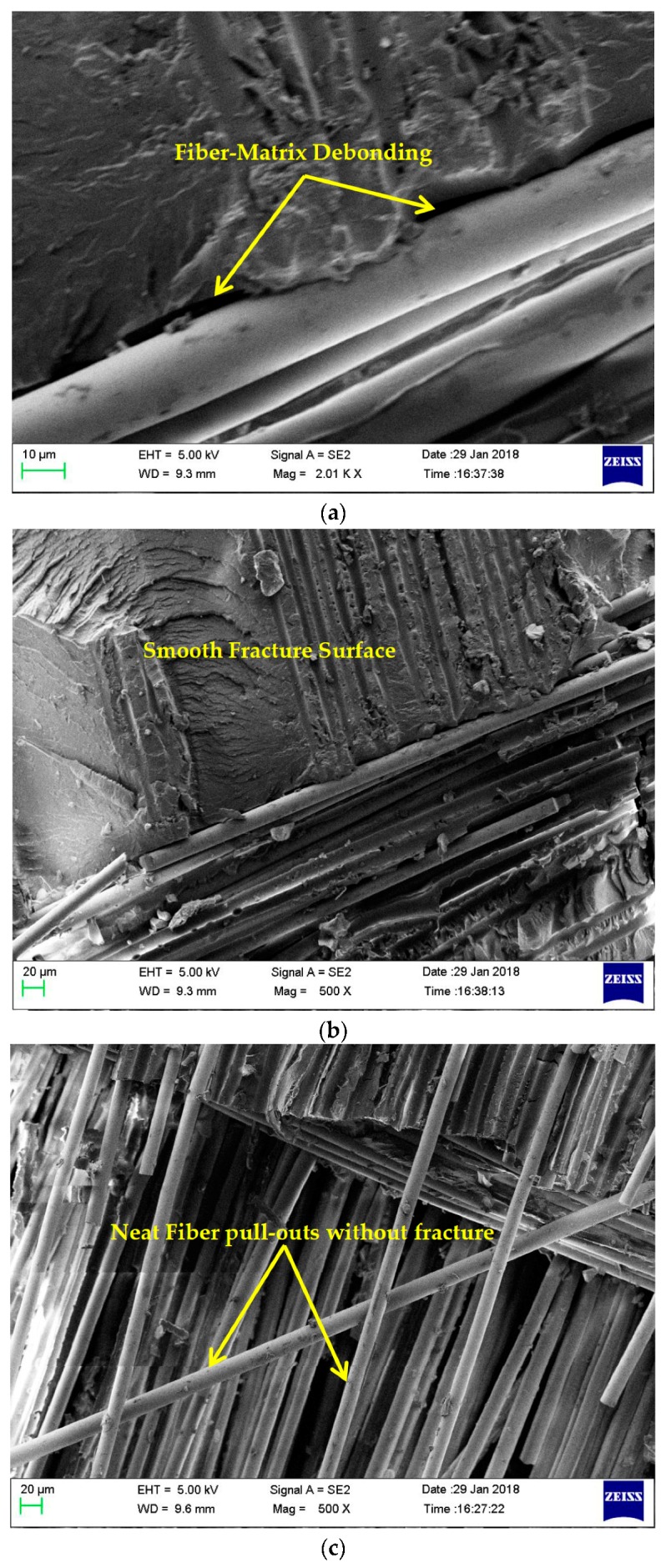
Failure surfaces of neat composites (**a**) poor interfacial bonding between matrix and fibre; (**b**) smooth fracture surfaces; (**c**) fibre pulled out neatly without fracture and damage.

**Figure 9:**
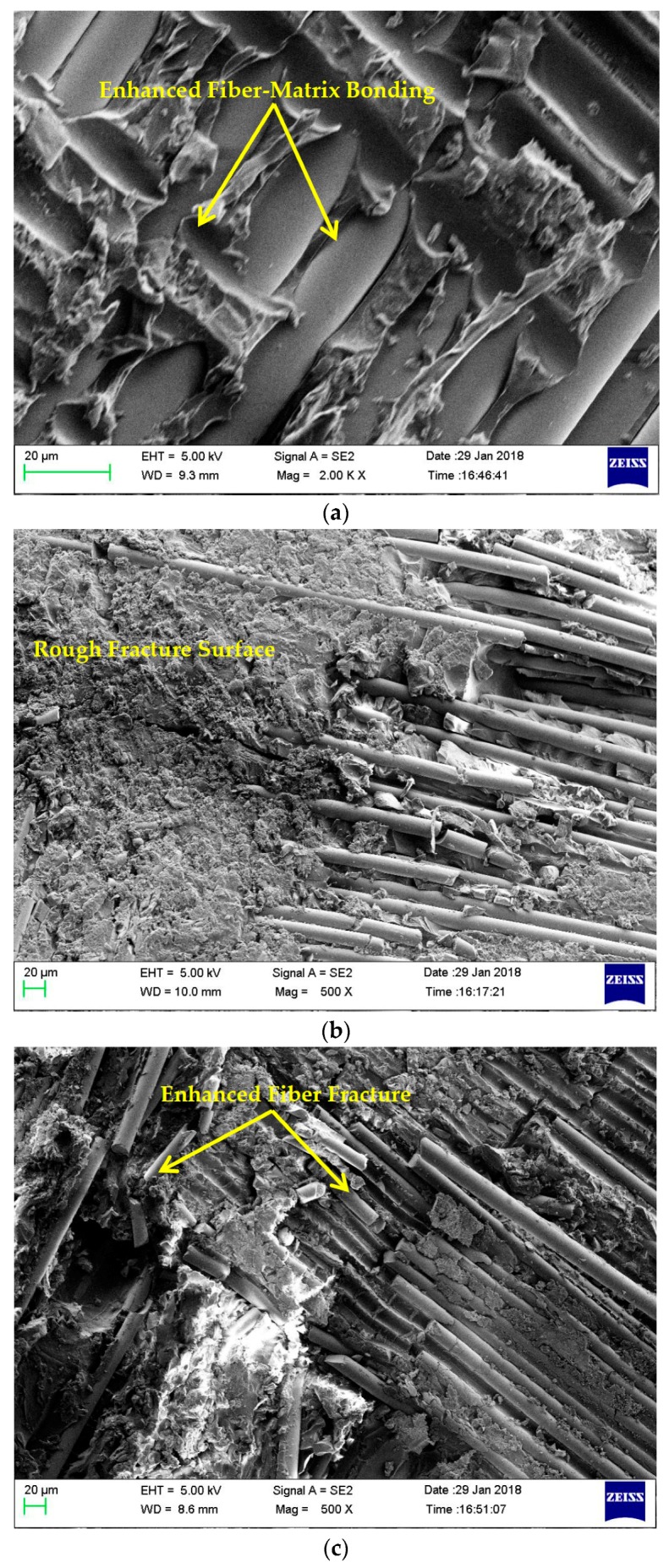

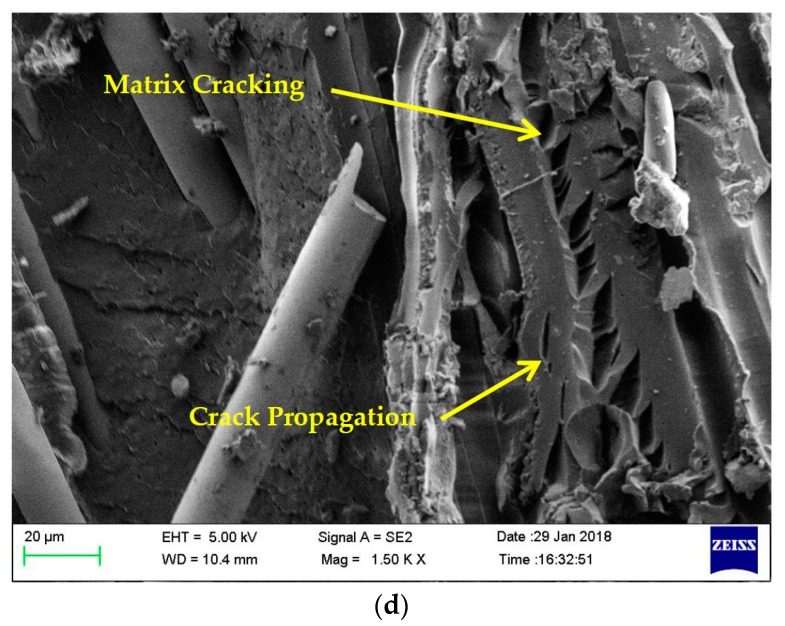
Fracture surfaces of 0.75 wt% nanocomposites (**a**) Enhanced fibre-matrix bonding; (**b**) Rough fracture surface; (**c**) Enhanced fibre-matrix damage; (**d**) Crack initiation and propagation.

To elucidate the effect of impregnating nanosilica in matrix, the fracture surfaces of the tensile specimens were analyzed using a ZEISS Scanning Electron Microscope (SEM, Olympus Corporation, Tokyo, Japan). The interfacial bonding and failure modes of neat composite laminates and laminates with 0.75 wt% nanosilica reinforced composite laminates were compared. The failure of matrix was induced by debonding of nano fillers and fibre from matrix [38]. It is considered to be the key toughening mechanism for nanosilica reinforced epoxy composites. Figure 8 presents the failure surfaces of neat composites. Figure 8a revealed the poor adhesion between matrix and fibre filaments in neat composites which ended up in weak interfacial bonding. From Figure 8b, it is evident that, unreinforced neat epoxy exhibited smooth fracture surface that evidenced the poor crack initiation and propagation. In Figure 8c, it is undoubtedly evident that fibre filaments of neat composite were pulled out from matrix without any fracture or damage. These indicate that, minimum stress was transferred by matrix to fiber in neat composites. Figure 9 presents the fracture surfaces of 0.75 wt% nanocomposites. Figure 9a highlights enhanced fibre-matrix bonding, Figure 9b,c portraits rough fracture surface and high fibre-matrix damages were witnessed. Multiple cracks were induced and propagated as highlighted in Figure 9d.

It is to be noted that new cracks were developed before the complete propagation of existing cracks. This was due to crack bowing mechanism [31], which was the toughening mechanism frequently noted in nano particle reinforced polymer materials. Moreover, the elongation at break was noted to increase to higher range due to the addition of silica nanoparticle ending up in enhanced stress transfer. It was implicit that the maximum stresses were transferred. Interfacial bonding and energy dissipation behaviour were superior due to the addition of silica nanoparticles upto 0.75 wt%. Higher concentrations of 1 and 3 wt% secondary reinforcements exhibited contrary effects in the performance of materials due to matrix embrittlement. 

#### 3.2.2. Internal Damage Due to Quasi Static Compression

**Figure 10:**
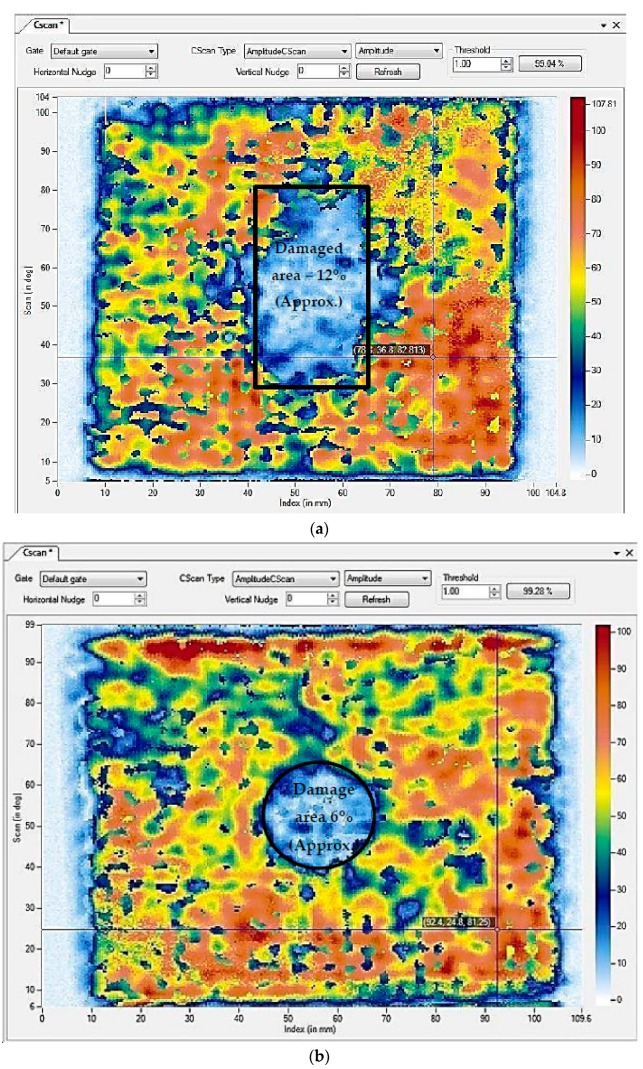
C-Scan results highlight detected surface damage in (**a**) neat composites; (**b**) 0.75 wt% nanosilica reinforced composites.

**Figure 11:**
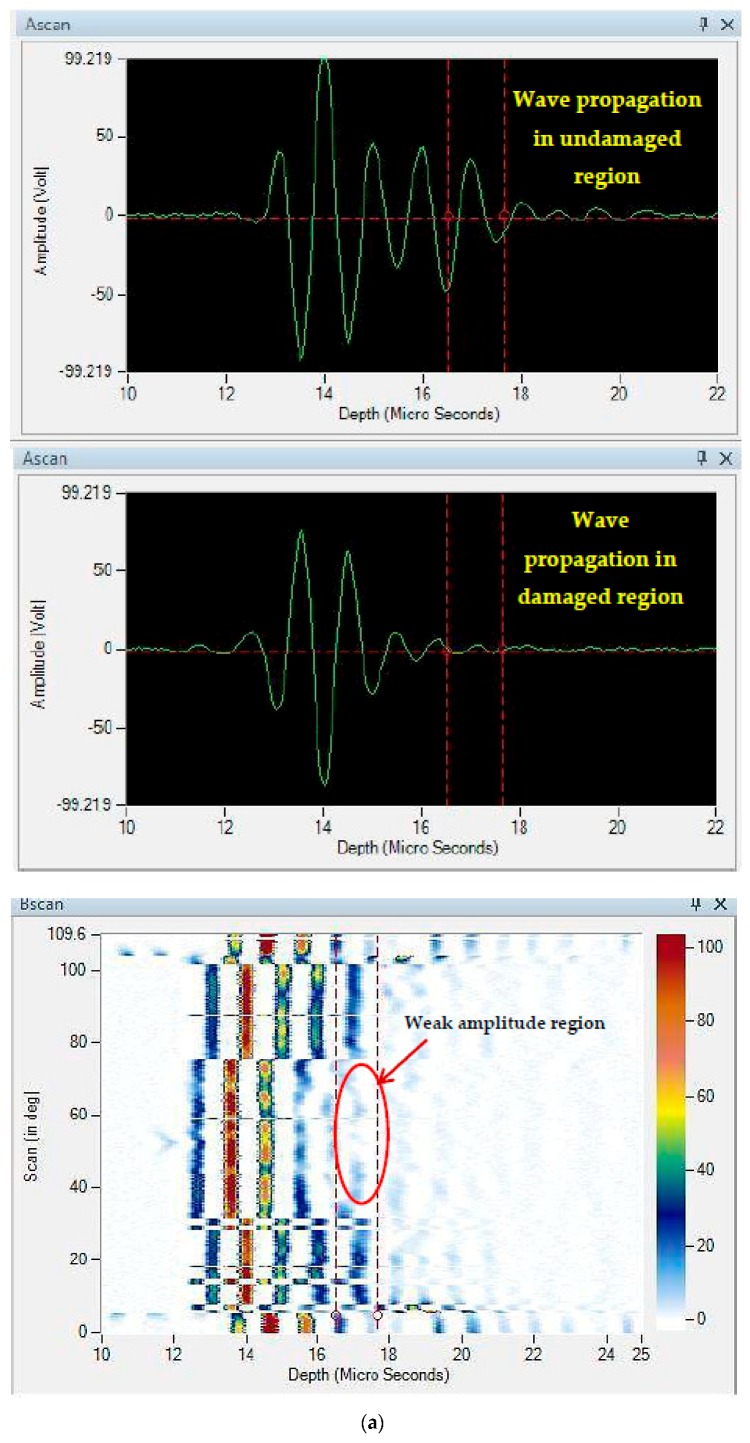

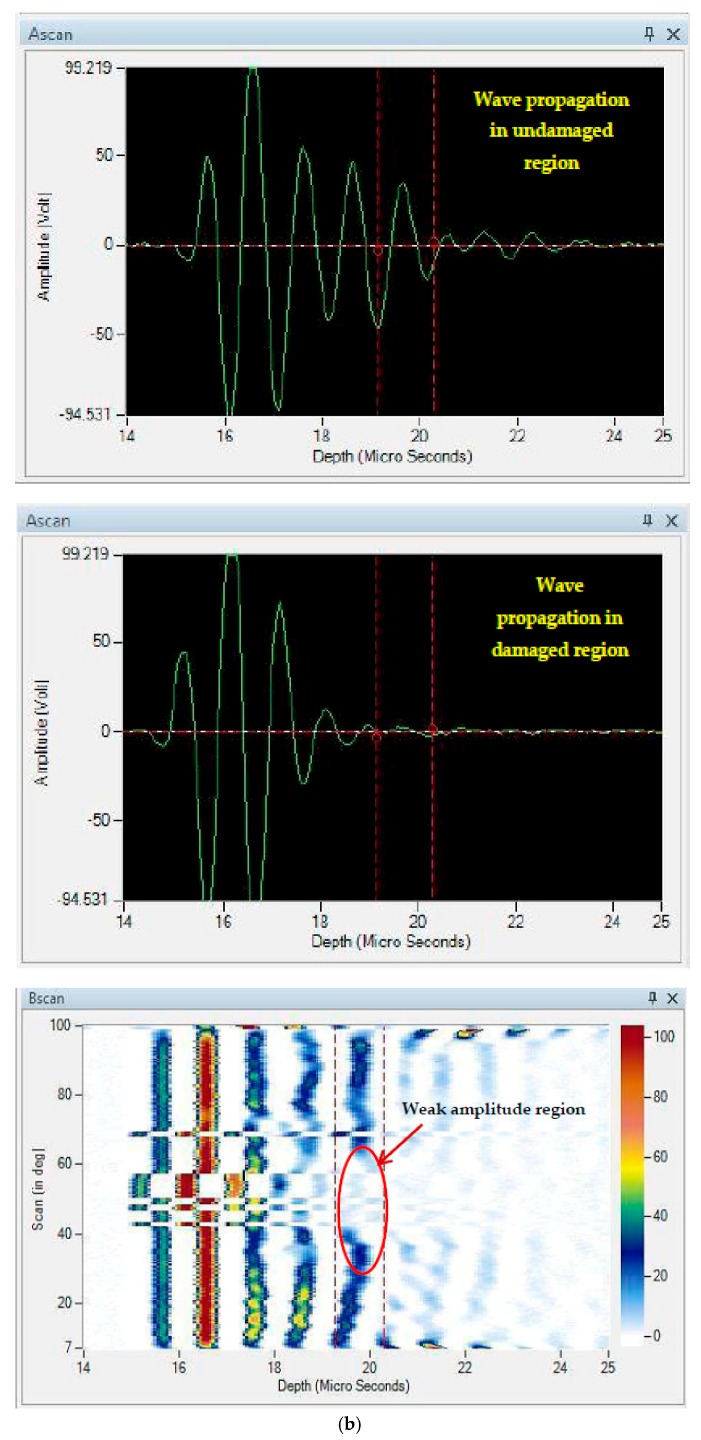
Loss of wave amplitude in (**a**) neat composite (**b**) 0.75 wt% nanosilica reinforced composite.

An advanced NDE technique C-Scan was used to understand the internal damage induced due to the quasi-static indentation tests. C-Scan results present the damage area along the plane. Figure 10 presents the NDE results of neat composite and 0.75 wt% nanosilica reinforced composites. From the results, it was detected that the damage was propagated to 12% in neat composites, whereas the damage was contained within 6% in 0.75 wt% nanosilica reinforced composite. Also the damage induced in nanosilica reinforced composites looks more predictable and propagates uniformly in all directions in a circular fashion. In contrast, damage in neat composites was largely distributed along a single axis providing a rectangular profile. The reason could be; delamination failure was propagated randomly along a weaker direction after matrix cracking. In terms of nano particles reinforced composites; the induced stresses were distributed evenly to the primary reinforcement through the secondary reinforcement. This could be; since secondary reinforcements enhanced the interfacial bonding of matrix and primary reinforcement as evidenced in SEM images, micro cracks propagated in all the directions instead of a single axis. These bred micro cracks had formed new micro cracks along their propagation and thus absorbed higher amount of energy and restricted the further propagation of failure. The loss of amplitude of ultrasonic waves in damaged and undamaged region is presented in A-Scan and B-Scan as shown in Figure 11. In neat composites, the amplitude of the waves was reduced drastically after 17 µs and no significant amount of signals were received further. Whereas, in nanosilica reinforced composites, amplitude of the waves was reduced drastically after 20 µs. This is due to the minimum damage induced through the thickness in the nanosilica reinforced composites. Results of A-scan and B-scan reveal that lower most layers of the composite laminates encounter the highest damage and the top most layers suffer with significant amount of damage.

## 4. Conclusions

Nanosilica particles were used as secondary reinforcement in epoxy matrix-glass fibre composite laminates and its effects on mechanical behaviour and damage behaviour were studied in detail. Results revealed that composite laminates reinforced with 0.75 wt% of nanosilica as secondary reinforcement registered a tensile strength of 295.4 MPa and a flexural strength of 346.12 MPa which is 42% and 39.46% higher than neat composites. In regard to visco-eleastic properties, loss modulus (E″) of nanosilica reinforced composites exhibited a decent increase when compared to neat composites. The highest value of loss modulus and tan δ was recorded by 0.75 wt% of nanosilica reinforced composite laminate. Also, noticed from quasi-static indentation tests, energy absorption of 15.32 Joules which was 57.93% higher than neat composites. SEM examinations reported poor interfacial bonding in neat composites and enhanced interfacial bonding in nanosilica reinforced composites. The propagation of multiple micro cracks, which is a significant failure mechanism, was also observed in nanosilica reinforced composites and the same was absent in neat composite. NDE results detected that, damage was contained within 6% along the failure plane and through the cross section in 0.75 wt% nanosilica reinforced composites whereas, it propagated till 12% in neat composites. From the assessments through mechanical properties, SEM and NDE, it is concluded that 0.75 wt% of nano silica is a potential candidate to be used as secondary reinforcement in epoxy matrix-glass fibre composite laminates.

## Figures and Tables

**Table 1:** Properties of plain weave E-glass fabric.

Property/Grade	E-Glass Fabric	Epoxy LY556
Density (g/cm^3^)	2.55	1.15
Tensile strength (MPa)	2000–3500	80–95
Tensile modulus (GPa)	70–73	0.3–0.6
Grade (GSM)	610	
Elongation at break (%)	2.5–3.7	

**Table 2:** Properties of Nanosilica.

Property	Value
Particle size (nm)	17
Specific surface area (m^2^/g)	202
Ph. value	4.12
Tamped density (g/L)	44
SiO_2_ content (%)	99.88

**Table 3:** Visco-Elastic properties of neat composites and nanosilica reinforced composites.

Laminate Identification	Nanosilica Content (wt%)	Glass Transition Temperature (T_g_)	Loss Modulus (MPa)	Damping Factor (tan δ)
DMA-L1	0	73.02	920	0.3347
DMA-L2	0.5	69.83	1188	0.455
DMA-L3	0.75	63.61	1261	0.499
DMA-L4	1	68.72	1008	0.426

**Table 4:** Quasi Static indentation properties of neat composite and nanosilica reinforced composites.

Sample No.	Nanosilica Content (wt%)	Compressive Stress (MPa)	Stiffness (MPa)	Peak Compressive Force (N)	Time to Peak Force (s)	Strain at Elastic Limit (mm)	Strain at Peak Force (mm)	Total Energy Till Peak Force (J)
QSI-L1	0	20.64	107.76	2984.49	71.3	4.01	5.99	9.7
QSI-L2	0.25	23.84	121.80	3447.76	77.05	4.489	6.433	12.01
QSI-L3	0.5	28.71	132.89	4152.15	83.4	5.18	6.923	14.03
QSI-L4	0.75	27.55	128.9	3984.43	85.3	5.411	7.116	15.32
QSI-L5	1	25.33	135.74	3662.62	83.3	5.196	6.874	13.42
